# Psychotherapy Works – An Inclusive and Affirming View to a Modern Mental Health Treatment

**DOI:** 10.32872/cpe.11971

**Published:** 2024-04-26

**Authors:** Christoph Flückiger, Ulrike Willutzki, Martin grosse Holtforth, Bruce E. Wampold

**Affiliations:** 1 Department of Psychology, University of Kassel, Kassel, Germany; 2 Department of Psychology, Witten/Herdecke University, Witten, Germany; 3 Department of Psychology, University of Bern, Bern, Switzerland; 4 Hospital Insel, Bern, Switzerland; 5 Department of Counseling Psychology, University of Wisconsin-Madison, Madison, WI, USA; Department of Psychology, University of Trier, Trier, Germany

**Keywords:** mental health, bona fide psychotherapy, transtheoretical psychotherapy, evidence-based psychotherapy

## Abstract

Psychotherapy is a highly collaborative and individualized mental health practice developed in (post-) modern societies. The mental health outcomes of psychotherapy cover a broad range of psychological factors including the reduction of suffering/symptoms as well as the promotion of well-being, personal values, and personal strengths. There is extensive meta-analytic evidence that legitimate psychotherapy works remarkably well and robustly for most common mental disorders. In addition, there is a large body of meta-analytic evidence supporting the potential relevance of transdiagnostic relationship principles and transtheoretical psychotherapy factors. Based on this ongoing empirical evidence, we propose four relevant implications for future training and practice in transdiagnostic psychotherapy: 1) the development of a transtheoretical legal framework for psychotherapeutic treatments, 2) the formulation of evidence-based transtheoretical interpersonal skills, 3) an orientation toward transtheoretical therapeutic factors, and 4) the exploration of comprehensive psychotherapy outcomes. We conclude with some more general guidance for future directions.

## Highlights

- Legitimate psychotherapy is remarkably effective.
- The evidence-based consolidation process on psychotherapy key principles is progressing continuously.
- There is robust empirical foundation of transtheoretical relationship factors and therapy skills.

There is comprehensive meta-analytic evidence that psychotherapy works across the most common mental health conditions. Surprisingly – and perhaps more controversially – there is robust evidence that various psychotherapy orientations work well, and when intended to be therapeutic (i.e., bona fide therapies) are approximately equally effective. That is, the ongoing controversy between psychotherapy orientations may make the potential differences between orientations appear larger than can be empirically supported, which is particularly true for long-term follow-ups. In this commentary, we provide explicit definitions of psychotherapeutic treatments and their mental health outcomes. Furthermore, we provide evidence-based examples of studies comparing the lasting efficacy at follow-up of particular legitimate psychotherapies vis-à-vis other legitimate psychotherapies. Next, we provide examples of evidence-based psychotherapy principles and skills based on recently conducted meta-analytic summaries. Last, we discuss implications for therapeutic practices and training, and conclude with some more general guidance for future directions.

## How Can Legitimate (Bona Fide) Psychotherapy Be Characterized and Why Is It Relevant to Identify Non Bona Fide Psychotherapy Conditions?

Psychotherapy is a highly collaborative and individualized mental health practice developed in (post-) modern societies (e.g., Elias, 1978; Huft, 2022). One of the main characteristics of psychotherapy is its collaborative nature. Furthermore, patients have a proactive role and are engaged in therapy. The key point is that psychotherapy is a socioculturally embedded mental health practice, with patients and society expecting a highly custom-tailored and collaborative talking cure to reduce target complains and fostering well-being and psychosocial functioning to proactively work on the clients´ sufferings.

One of the major challenges in psychotherapy research is that psychotherapy and its minimal standards are often not explicitly defined. We define the minimal standards of legitimate (bona fide) psychotherapy as follow (Wampold et al., 1997; Wampold & Imel, 2015): Psychotherapists with at least a master's degree provided treatment, and two of the following criteria are required: (a) treatment is generally recognized as legitimate psychotherapy, such as CBT or psychodynamic therapy, and therapists are not prohibited from using recognized therapeutic interventions, such as psychoeducation, empathy, a rationale for treatment, promotion of coping skills, or (b) the description of treatment includes a reference to a psychological mechanism (e.g., operant conditioning), or (c) a manual/guide is used, or (d) the treatment includes an active component that has appeared in the psychological literature. Careful consideration of the minimum standards of legitimate psychotherapy is a prerequisite for including only such trials in meta-analyses of psychotherapy comparisons to minimize research bias and triviality of findings.

With respect to *non bona fide* psychotherapy conditions, such conditions often are constructed to not be fully therapeutic (e.g., Westen et al., 2004; Munder et al., 2019; Flückiger et al., 2022). Non bona fide psychotherapy conditions often can be identified by looking at what interventions are excluded or even “banned”. Examples of non bona fide psychotherapy in the above-mentioned sense are: *discussion group* where the leaders were instructed to “not teach skills or differentially reinforce coping strategies” (Wetherell et al., 2003, p. 33), *psychoeducation* where “instructors were asked, as best as they could, not to teach any skills in a way that may enhance mindfulness” (Wong et al., 2016, p. 69), *nondirective therapy* where “direct suggestions, advice or coping methods were prohibited” (Borkovec & Costello, 1993, p. 613), three session *contact control group* to motivate patients “to wait for the start of the treatment” (Linden et al., 2005, p. 37).

In many cases such non bona fide “intent-to-fail”-interventions are used to demonstrate the relevance of specific components of the favored treatment (Westen et al., 2004). However, these conditions are not informative about the efficacy of the approach compared to legitimate psychotherapy and provide little information about specific ingredients.

## How Can Psychotherapy Outcomes Be Defined?

WHO defines mental health as follows:

*Mental health is a comprehensive state of mental well-being that enables people to cope with the stresses of life, realize their abilities, learn well and work well, and contribute to their community. It is an integral component of health and well-being that underpins our individual and collective abilities to make decisions, build relationships and shape the world we live in. (World Health Organization, WHO, 2022, June 17).*

Consistent with the above-mentioned broad definition of the World Health Organization (WHO), we understand mental health as a lifelong process of development and adaptation. Most importantly, mental health has at least two constituents, including the reduction of suffering/symptoms as well as the promotion of well-being, personal values, and strengths (e.g., Schürmann-Vengels et al., 2022). In psychotherapy, collaborative efforts toward symptom reduction and well-being represent psychotherapeutic outcomes that result from careful therapeutic exploration (grosse Holtforth et al., 2004). The point relevant to this Special Issue on transtheoretical clinical training and practice is that psychotherapy outcomes go beyond the tailored ("primary") outcomes of a particular psychotherapy and should cover the broad spectrum of the WHO definition of mental health.

## Does a Particular Bona Fide Psychotherapy Have a More Lasting Effect Than Another?

Although somewhat arbitrary in nature, a threshold for a clinically relevant treatment effect in anxiety and mood disorders has been estimated to be about *d* = .25 (Cuijpers et al., 2014). Comparative meta-analyses report small to negligible relative differences in efficacy if two or more bona fide psychotherapies are compared directly in an RCT design (Wampold & Imel, 2015).

With respect to long-term outcomes, Kivlighan and co-authors (2015) for example examined the long-term follow-ups of bona fide psychodynamic psychotherapies compared with non-psychodynamic bona fide psychotherapies (*k* = 25). They hypothesized that psychodynamic psychotherapies would perform better over time than other treatments (assuming a positive and statistically significant slope effect from assessment post-therapy to follow-up). However, they found an effect of *d*_slope_ = .00 for disorder-specific targeted outcomes, nontargeted outcomes, and personality outcomes. These results suggest that the efficacy of psychodynamic therapies is generally not more sustainable but also not less sustainable after active treatment compared with other bona fide psychotherapies. Several other longitudinal multilevel meta-analyses on interventions for anxiety and depression did not show any significant increase or decrease in relative effects over follow-up time within studies, such as for cognitive interventions versus behavioral interventions (Podina et al., 2019), studies with additive components (Flückiger et al., 2015), and established cognitive behavioral therapy or augmented integrative cognitive behavioral therapy for generalized anxiety disorder under bona fide conditions (Flückiger et al., 2022; for all studies *d*_slope_ < .10). Again, these meta-analytic findings show that differential long-term outcomes for particular bona fide psychotherapy approaches usually are not likely.

The point relevant to the Special Issue for transtheoretical training and practice is that the enormous effort to specify psychotherapeutic effects through very specific approach explains surprisingly little on the long run. Do we therefore face a shambles? No, we think not! On the contrary, as we will show in the following sections there is considerable meta-analytic evidence for the outcome-relevance of multiple transtheoretical principles and skills.

## Is There an Evidence Base for Transtheoretical Psychotherapy Principles and Skills?

Psychotherapy represents a cooperative course of action between therapist and patient during and between psychotherapy sessions. One of the most substantiated findings in psychotherapy research is that transtheoretical collaborative qualities are robustly linked to treatment outcomes across many psychotherapy conditions. Based on an international meta-analytic summary of 295 studies representing more than 30,000 psychotherapies, there is a moderate predictive association of 8% explained variance (*r* = .278; confidence interval .256 ≤ *r* ≤ .299; Flückiger et al., 2018) between alliance measured mostly once during therapy and therapy outcome (at the end of therapy) in face-to-face and internet-mediated treatments. These statistically significant results confirm those of previous meta-analyses. A comparable predictive power of the working alliance could also be meta-analytically confirmed in therapies with children and adolescents (Karver et al., 2018), in couple and family settings (Friedlander et al., 2018) and groups (Lo Coco et al., 2022).

There seem to be therapists who are comparatively more successful in building working alliances with their patients than others. These differences between therapists are relevant for treatment success, i.e., therapists who on average build better alliances also treat somewhat more successfully. These moderate effects have been meta-analytically confirmed (Del Re et al., 2021).

It is hypothesized that the alliance-outcome relationship manifests itself in particular because of the patients' intake characteristics (e.g., Feeley et al., 1999). However, based on 66 studies reporting both uncontrolled predictor models and predictor models controlled for the intake variables, there is no systematic evidence that the alliance can be fundamentally understood as an epiphenomenon of the intake variables, arguing for the collaborative conception *during* treatment (*r* = .25 vs. .22; Flückiger, Del Re, et al., 2020). Furthermore, an individual participant data analysis of 17 studies indicated that in the early phase of therapy, symptoms and alliance were reciprocally related to one other, often resulting in a positive upward spiral of higher alliance/lower symptoms that predicted higher alliances/lower symptoms in the subsequent sessions (Flückiger, Rubel, et al., 2020). Overall, the available evidence across hundreds of studies indicates that the above-mentioned sociocultural key principle of psychotherapy as a highly collaborative mental health treatment is robustly linked to psychotherapy outcomes. For other concepts that partly overlap with the alliance concepts comparable correlation patterns have been shown (for example empathy, goal agreement and group cohesion; Norcross & Lambert, 2018).

With respect to particular therapist skills, a recent APA interdivisional effort investigated 27 well-accepted basic psychotherapy skills and methods (Hill & Norcross, 2023). The results of the meta-analytic summaries revealed that some transtheoretical psychotherapy skills such systematic feedback in routine outcome monitoring (Barkham et al., 2023), emotion-regulation strategies (Iwakabe et al., 2023) or strength-based methods (Flückiger et al., 2023) were evaluated as “demonstrably effective” for post-treatment outcomes (published open access in the journal *Psychotherapy Research;* Hill & Norcross, 2023). Overall, findings from process-outcome research have the potential to moderately improve psychotherapy interventions across psychotherapy orientations.

## Clinical and Training Implications for Future Training and Practice in Transtheoretical Psychotherapy

Based on the reviewed literature, the following transtheoretical, transdiagnostic and interdisciplinary implications can be summarized for future clinical practice and training.

### Development of a Legal Framework for Transtheoretical Mental Health Treatments and Psychotherapy

We consider it a key societal achievement that legislators have prioritized mental health treatments that have a strong collaborative foundation. Coercive measures are used only in extreme emergencies. Psychotherapy is the best example of how the joint negotiation and decision-making process can be carefully elaborated within mental health systems. The alliance, as one of the most studied collaborative principles, emphasizes the overall meaning of therapy, which includes therapeutic goals, therapeutic tasks, and deeper bonds of trust in the confidentiality of psychotherapy. Legitimate psychotherapy fundamentally requires consensual collaboration between therapist and patient.

The relevant point for training and practice is that psychotherapy provides a socially protected setting where patients are allowed to express their innermost concerns and desires. Psychotherapy is a cultural practice that promotes humane and free society. Psychotherapy does not exclude people and brings individuals, couples, families and groups together. It offers understanding for diversity, cultural sensitivity, psychosocial exclusion and psychological strain.

### Evidence-Based Transtheoretical Interpersonal Skills

Building on patients’ alliance potential and enhancing their alliance qualities at the beginning of a therapy is, on the one hand, central to ensuring that patients do not immediately discontinue the therapy. On the other hand, the early alliance (among other factors) lays the foundation for patients to engage in tasks of therapy. In the first phase of therapy, it is crucial that the methods of therapy are tailored to the patient's specific expectations, skills and abilities, and needs. The collaborative qualities of psychotherapy are crucial, namely that patients and therapists basically agree independently to the joint therapeutic tasks. The quality of the alliance may fluctuate within sessions. In principle, central tendency is more relevant to success than a single session. However, critical, negative to hostile reactions from patients are possible during sessions and interruptions of the alliance are not uncommon. Adaptation of procedures to the patients’ motives and strengths can enhance the alliance quality. In addition, tears and ruptures in the therapeutic relationship can be explored in a non-catastrophizing way and, if necessary, used therapeutically (Eubanks et al., 2023; also Caspar & grosse Holtforth, 2009; Safran & Muran, 1996).

### Navigating Transtheoretical Therapeutic Factors

Navigating and monitoring the patients´ views of psychotherapeutic factors is an important therapist task to ensure that therapists do not take them for granted too quickly or become overly critical of themselves and the therapy. Therapists differ in the quality of how they build and shape custom-tailored treatments. What basically counts is the overall collaborative quality of the therapeutic process and not the adherence to an ostensible therapeutic stereotype.

### Broadening Psychotherapy Outcomes

As the above-mentioned health definition exemplifies, mental health is a much more comprehensive condition than the absence of particular symptoms and related disorders. At the same time, well-being is not an exclusive goal for psychotherapeutic treatments. Singing in a choir, sailing with friends, or listening to a punk rock band can similarly contribute to mental health depending on the individuals´ preferences. Clearly, such activities may be highly relevant topics in the consolidation phase of a therapy to explore psychotherapeutic change. The critical point is that a wide range of mental health measures need careful consideration when evaluating mental health treatment.

**Figure 1:**
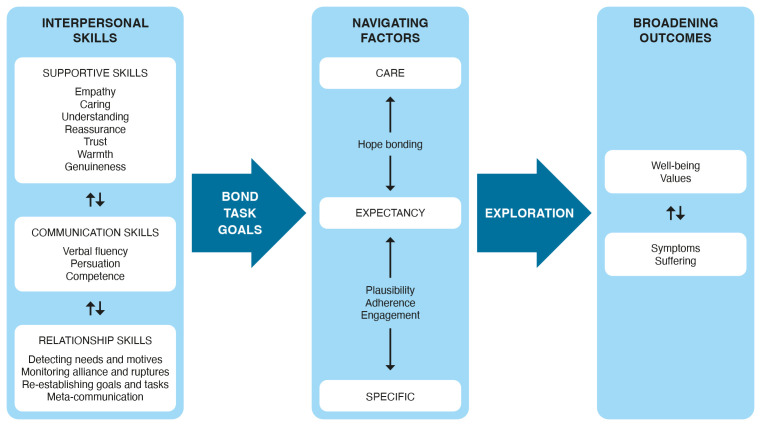
Exemplification of an Evidence-Based Transtheoretical Model – The CARE-Model of Mental Health Treatments (Adapted From Wampold & Flückiger, 2023)

To promote effective learning and therapy practice, supportive psychotherapy training (e.g., fostering supportive supervision, positive trainee relationships, and group collaboration; Heinonen et al., 2022) as well as structured practice on basic skills (e.g., Anderson et al., 2020; Eells et al., 2005) are essential. We expect a continuing psychotherapy development of therapists over a career that comprises professional and personal progression as well as challenges (Orlinsky et al., 2005). Figure 1 shows an example of how an evidence-based transtheoretical model might be formulated (see Wampold & Flückiger, 2023), which is an extension of Contextual Model (Wampold & Imel, 2015). In the transtheoretical model, there are three pathways to the benefits of all mental health (as well as physical health) service: The CARE pathway (caring, attentive, real, empathic), the Expectation pathway, and the Specific pathway. In a sense, this model integrates the effects of relationship factors and specific ingredients, making it important for all psychotherapists, including those strongly affiliated with a particular treatment, as well as for various healing domains, including psychotherapy, psychiatry, and medicine. The model is also transcultural as well as transtheoretical.

## Concluding Comment for Future Directions

Mental health is a human right and a shared responsibility of societies that cannot be entirely delegated to particular professions nor achieved by certain treatments. At the same time, carefully conducted mental health treatments are cultural achievements. Transtheoretical therapeutic factors such as collaborative qualities are relevant across orientations but also across professions and settings (Wampold & Flückiger, 2023). On the healthy side of mental health, there is openness, individuality, and basic human freedom in how we create our lives. Accepting co-responsibility for psychotherapeutic outcomes is not only a critical interpersonal skill of therapists, but also entails an attitude of trust in joint exploration of what individual well-being means.
